# Metabolic effect of TAp63α: enhanced glycolysis and pentose phosphate pathway, resulting in increased antioxidant defense

**DOI:** 10.18632/oncotarget.2300

**Published:** 2014-07-31

**Authors:** Angelo D'Alessandro, Ivano Amelio, Celia R. Berkers, Alexey Antonov, Karen H. Vousden, Gerry Melino, Lello Zolla

**Affiliations:** ^1^ Department of Ecological and Biological Sciences, University of Tuscia, Largo dell'Università, snc, Viterbo, Italy; ^2^ Department of Biochemistry and Molecular Genetics, University of Colorado Denver – Anschutz Medical Campus, Aurora, CO, USA; ^3^ Medical Research Council, Toxicology Unit, Hodgkin Building, Leicester University, Lancaster Road, Leicester, UK; ^4^ CR-UK Beatson Institute, Switchback Road, Glasgow, UK; ^5^ Current address: Bijvoet Center for Biomolecular Research, Utrecht University, Padualaan 8, Utrecht, The Netherlands; ^6^ Biochemistry Laboratory IDI-IRCCS, c/o/ Department of Experimental Medicine and Biochemical Sciences, University of Rome “Tor Vergata”, Via Montpellier 1, Rome, Italy; ^7^ Institute of Cellular Biology and Neurobiology, CNR, Rome, Italy

**Keywords:** p63, p53 family, metabolomics, glycolysis, glutaminolysis

## Abstract

TAp63α is a member of the p53 family, which plays a central role in epithelial cancers. Recently, a role has emerged for p53 family members in cancer metabolic modulation.

In order to assess whether TAp63α plays a role in cancer metabolism, we exploited p53-null osteosarcoma Tet-On Saos-2 cells, in which the expression of TAp63α was dependent on doxycycline supplementation to the medium. Metabolomics labeling experiments were performed by incubating the cells in ^13^C-glucose or ^13^C^15^N-glutamine-labeled culture media, as to monitor metabolic fluxes upon induced expression of TAp63α.

Induced expression of TAp63α resulted in cell cycle arrest at the G1 phase. From a metabolic standpoint, expression of Tap63α promoted glycolysis and the pentose phosphate pathway, which was uncoupled from nucleotide biosynthesis, albeit prevented oxidative stress in the form of oxidized glutathione. Double ^13^C-glucose and ^13^C^15^N-glutamine metabolic labeling confirmed that induced expression of TAp63α corresponded to a decreased flux of pyruvate to the Krebs cycle and decreased utilization of glutamine for catabolic purposes in the TCA cycle. Results were not conclusive in relation to anabolic utilization of labeled glutamine, since it is unclear to what extent the observed minor TAp63α-dependent increases of glutamine-derived labeling in palmitate could be tied to increased rates of reductive carboxylation and *de novo* synthesis of fatty acids. Finally, bioinformatics elaborations highlighted a link between patient survival rates and the co-expression of p63 and rate limiting enzymes of the pentose phosphate pathway, G6PD and PGD.

## INTRODUCTION

Decades after Warburg's pioneering observations [1], cancer metabolic reprogramming has come to be considered a key driver of tumor progression [2] and a so-called “hallmark of cancer” [3-5]. Malignant transformation is indeed driven by the sequential acquisition of mutations to oncogenes and tumor suppressor genes, but this is strictly tied to metabolic reprogramming in the light of the increasingly documented influence of oncogenes and tumor suppressor genes on cell metabolism [6-10]. In this view, a central role has recently emerged for p53 and its family members (p63 and p73) in the modulation of several metabolic pathways [9]. Tp53-family members are supposed to maintain cell energy and redox poise in response to external stimuli, including genotoxic stresses, in a “save or sacrifice” fashion [9]. Activation of p53 might also trigger cell death or senescence in response to irreversible damage, such as extreme genotoxic damage or the activation of oncogenes [9].

The gene structure of p53-family tumor proteins (TP53, TP63 and TP73) is characterized by three conserved domains [11], including the N-terminal transactivation domain (TA), the central DNA binding domain (DBD), and the C-terminal oligomerization domain. Owing to the presence of two distinct promoters, TP63 and TP73 may utterly result in two diametrically opposing classes of proteins: those containing the N-terminal TA domain (TAp63 and TAp73) and those lacking it (ΔNp63 and ΔNp73), each one characterized by distinct and sometimes opposite biological functions [12].

In like fashion to p53, TA-proficient isoforms of both p63 and p73 have been implicated in tumor suppression and protection from metastasis (especially in invasive bladder and prostate cancer) [11]. Moreover, p63 expression is absolutely essential for limb formation and epidermal morphogenesis (integument and tongue) including the formation of adnexa (teeth, hair, mammary and prostate glands, and sweat and lacrimal glands), skin and most epithelial tissues (e.g., prostate and mammary glands), while p63-null animals – surviving only a few days post-natally - show severe limb truncations or absence of limbs and skin, in addition to craniofacial malformations [11].

Metabolic effects of Tap63-isoforms have been anticipated, in analogy to p53 and Tap73. Indeed, although p53 plays a well-established role in the inhibition of cancer development, it has also been shown to promote cell survival in response to transient or mild stress (such as nutrient deprivation [13]) by triggering cell cycle arrest and boosting anti-oxidant defenses, as to decrease ROS levels and allows the cell to cope with DNA damage. For example, Tp53 downstream targets include Synthesis of Cytochrome c Oxidase 2 (SCO2), a critical regulator of the cytochrome c oxidase complex [14], and TP53-induced glycolysis and apoptosis regulator (TIGAR) [15]), promoting a build-up of early glycolytic intermediates and a shift towards the pentose phosphate pathway (PPP). In this view, another key target of Tp53 is glutaminase 2 (GLS2) [16-18], an enzyme involved in glutamine metabolism and, indirectly, in the regulation of glutathione homeostasis and glutamine-derived glutamate fluxes through the Krebs cycle. More recent findings have identified carnitine palmitoyltransferase 1C (CPT1C) as a novel target of p53 [19]. CPT1C is a brain-specific member of a family of mitochondria-associated enzymes that play a central role in fatty acid metabolism. CPT1C anti-correlates with mammalian target of rapamycin (mTOR) pathway activation, and promotes cell survival and tumor growth [20]. In fact, CPT1C expression is triggered by metabolic stress factors, such as hypoxia and glucose deprivation, in a p53 and AMP activated kinase-dependent manner [20]. Finally, in addition to its nuclear localization, the p53 protein has been also detected on the mitochondria, and here it seems to be able to affect cell respiration as well as interacting with the mitochondrial F_1_F0-ATP synthase[21].

TAp73 has been implicated in autophagy, the control of reactive oxygen species (ROS), and the maintenance of mitochondrial complex IV [22], other than the induction of endoplasmic reticulum (ER) stress, activation of the pentose phosphate pathway (PPP) [23] and alteration of serine biosynthesis [24].

On the other hand, Tap63 might have unique effect on cancer cell metabolism. For example, proteomics and targeted metabolomics have suggested a peculiar role of TAp63 isoforms in enhancing glycolysis [25]. In addition, p63 influences lipid metabolism, by increasing fatty acid synthesis and decreasing fatty acid oxidation [26].

In 2006, Gressner and colleagues generated a Tet-On-inducible osteosarcoma (Saos-2) cell line, expressing TAp63α in response to doxycycline supplementation to the medium. This cell model lends itself to investigate the biological role of TAp63α, since Saos-2 cells are p53 negative and show no detectable p63 and p73 at either mRNA or protein level [27]. The expression of TAp63α protein resulted in the induction of p21, and was accompanied by cell cycle arrest at the G1 phase and the onset of apoptosis [27]. Apoptosis was mediated by the TAp63α-triggered expression of the CD95, TNF-R and TRAIL-R death receptors [27]. Furthermore, TAp63α induced apoptosis via the mitochondrial pathway, through the up-regulation of caspases and pro-apoptotic Bcl-2 family members, like Bax and BCL2L11, and the expression of RAD9, DAP3 and APAF1 [27]. In order to gain further insights on TAp63-dependent metabolic modulation, in the present study Tet-On TAp63α Saos-2 cells were cultured in the presence of media either containing ^13^C-glucose or ^13^C ^15^N-glutamine stable isotopes. While heavy glucose is the most common metabolic tracer, in the light of the generality of the Warburg effect, the advantages of monitoring cancer cell utilization of glutamine are related to the emerging role of glutamine metabolism in cancer cells [28,29]. This is in keeping with our recent evidence that, in analogy to p53 and p73 [16,17,24], p63 can modulate the expression of GLS2 [30].

Our data indicate that TAp63α causes a large series of metabolic modulations, involving central carbon metabolism (glycolysis, pentose phosphate pathway, nucleotide biosynthesis). The present results also suggest that serine and fatty acid catabolism/biosynthesis might be affected by Tap63α. In induced cells, catabolism of glutamine-derived glutamate through the TCA cycle was slower in comparison to non-induced controls. These data are relevant to further understand the role of p63 in cancer.

## RESULTS AND DISCUSSION

### TAp63α promotes glycolysis and PPP pathway, uncoupled from nucleotide biosynthesis

**Figure 1:**
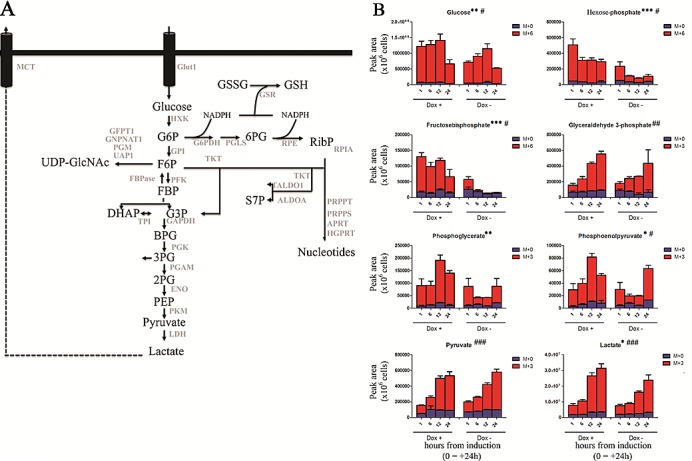
Effect on glycolysis (A). A simplified overview of glycolysis, pentose phosphate pathway and hexosamine pathway. Abbreviated metabolites and enzymes (Uniprot names) are reported in black and gray, respectively. (B) Isotopomer distribution of metabolites of glycolysis, graphed from left to right, from top to bottom in their order of appearance in the Embden-Meyerhoff pathway. Grouped columns in the left indicate TAp63α-expressing (doxycyline supplemented cells – Dox +), while columns in the right refer to non-induced controls (Dox -). Isotopomers are indicated as M+0 (only light unlabeled ^13^C atoms), M+6 (for hexoses deriving all six carbon atoms from heavy glucose) and M+3 (heavy trioses). Kinetics assays have been performed by harvesting cells at 1, 6, 12 and 24h, from medium replacement (which in turn was performed at 24h from doxycyline supplementation). * and # indicate statistical significance for inter-group (Dox + vs Dox -) or intra-group variations (time-course analyses). The number of * and # is related to *p-values* < 0.05; 0.01 or 0.001, respectively.

To investigate the metabolic function of TAp63, we employed a Tet-On inducible SaOs-2 osteosarcoma cell line, carrying the inducible expression vector coding for a HA-tagged TAp63α. TAp63α induction was functional as it promoted the expression of the direct transcriptional target p21 (Suppl. Fig. 1A) resulting in a G1 cycle arrest (Suppl. Fig. 1B), consistently with the literature[27]. To assess whether TAp63 has any regulatory effects on the glucose flux, we induced Tap63α expression by supplementing doxycycline to the medium. After 24h we replaced cell media with DMEM containing ^13^C-glucose stable isotopes. MS analysis revealed that glucose consumption via glycolysis was increased in TAp63α expressing cells compared to untreated controls. In particular, intracellular labeled glucose was significantly higher in doxycycline-supplemented units (Fig. 1B). In addition induced expression of TAp63α corresponded to a statistically significant accumulation of early (M+6) and late (M+3) glycolytic intermediates (Fig. 1B). This increase of glycolytic rate also corresponded to a faster rate of intracellular lactate accumulation (Fig. 1B). These results are consistent with recent proteomics and targeted metabolomics evidence about a role of TAp63 isoforms in enhancing glycolysis in colon cancer stem cells[25].

**Figure 2:**
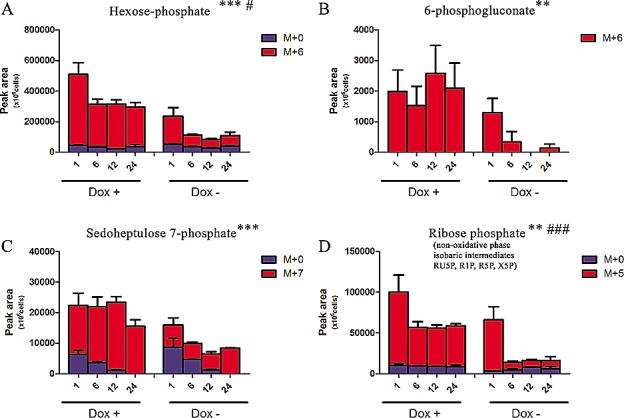
Effect on Pentose Phosphate Pathway Isotopomer distribution of metabolites of the Pentose Phosphate Pathway, graphed from A to D in their order of appearance (oxidative and non-oxidative arms). Grouped columns in the left indicate TAp63α-expressing (doxycyline supplemented cells – Dox +), while columns in the right refer to non-induced controls (Dox -). Isotopomers are indicated as M+0 (light, no heavy ^13^C atoms incorporated), M+6 or M+5 (for hexoses and pentoses deriving all their carbon atoms from heavy glucose). Kinetics assays have been performed by harvesting cells at 1, 6, 12 and 24h from medium replacement (which in turn was performed at 24h from doxycyline supplementation). * and # indicate statistical significance for inter-group (Dox + vs Dox -) or intra-group variations (time-course analyses). The number of * and # is related to *p-values* < 0.05; 0.01 or 0.001, respectively.

Increased glycolysis might be also associated to increase of anabolic branch pathways derived from glycolysis (Fig. 1A). Therefore we next investigated whether the accumulation of glycolytic intermediates was associated to an increase in refueling of pentose phosphate pathway (PPP). Induced-expression of TAp63α indeed was also accompanied by the accumulation of labeled PPP intermediates, both of the oxidative phase (glucose 6-phosphate, 6-phosphogluconate) and non-oxidative phase (sedoheptulose 7-phosphate and ribose phosphate – and isobaric non-oxidative PPP compounds) (Fig. 2A-D), indirectly suggestive of a PPP-mediated increase in NADPH/NADP^+^ ratios. On the other side, the TAp63α-dependent increase of PPP was compensated by a downregulation of the potentially pro-oxidant hexosamine pathway, as gleaned through double metabolic labeling (Suppl. Fig. 2A,B).

Despite higher accumulation of labeled ribose (Fig. 2D), slower incorporation of heavy ribose moiety was observed in purine nucleotides in ^13^C-glucose experiments (Suppl. Fig. 3A), suggestive of a likely enzymatic blockade downstream to ribose. Further steps of nucleotide *de novo* synthesis were deregulated in response to the expression of TAp63α, as confirmed through 13C15N-glutamine labeling experiments (Suppl. Fig.3.B). Impaired nucleotide biosynthesis was consistent with the previously reported[27] and hereby confirmed TAp63α–depdendent cell cycle blockade at the G1 phase and decrease in S phase (Suppl. Fig. 1B).

### Effect of TAp63α on serine and GSH homeostasis

Serine levels are relevant in cancer cells [*for review Amelio et al.*[31], since serine influences pyruvate kinase M2 (PKM2) activity, and thus the glycolytic rate of the cell and *de novo* biosynthesis of serine itself [32]. The efficiency of this pathway is regulated under serine starvation in a p53/p21-dependent[13] and p73-dependent fashion [24]. Serine anabolism also fuels the biosynthesis of other key aminoacids, such as glycine and cysteine, through transamination reactions that contribute to the cellular anaplerotic flux of glutamine to glutamate. Cysteine, glycine and glutamate can thus promote GSH anabolism, or fuel tricarboxylic acid (TCA) cycle via glutamate-derived α-ketoglutarate[33,34]. From a metabolomics standpoint, serine light isotopomers (either uptaken from the supernatant or deriving from proteolysis) progressively decreased both in doxycycline-supplemented and untreated controls (Suppl. Fig. 4). Labeling experimental approaches either using ^13^C-glucose or ^13^C^15^N-glutamine revealed a TAp63α-dependent accumulation of heavy labeled serine (Suppl Fig. 5). This can either derive from a reduced consumption of serine in induced cells, or from a TAp63α-dependent stimulation of serine biosynthesis similarly to the family members p53[32] and TAp73[24]. Conversely, conversion of serine in glycine appeared reduced, consistently with a likely minor serine consumption in induced cells. Indeed, serine-derived glycine biosynthesis was slower in doxycycline-treated cells compared to controls both in ^13^C-glucose and ^13^C^15^N-glutamine labeling experiments (Suppl. Fig. 5B-E). Analogously, heavy labeled cystathionine (an intermediate of cysteine biosynthesis from serine and homocysteine) was lower in TAp63α overexpressing cells (Suppl. Fig. 5C-F).

**Figure 3:**
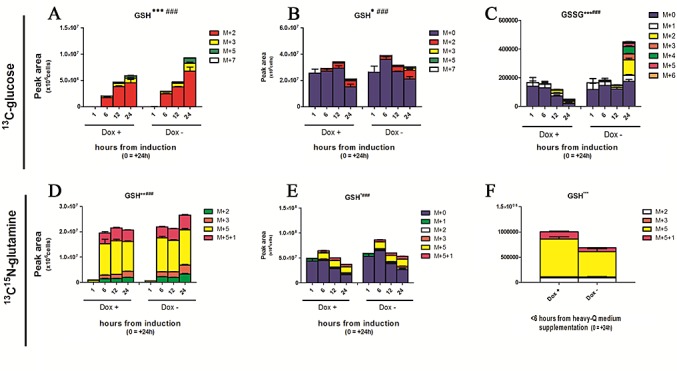
Effect on GSH homeostasis (A-F) Heavy and total isotopomers of reduced and oxidized glutathione (GSH and GSSG, respectively), as representative metabolites for oxidative stress accumulation. In A, heavy isotopomers are reported on the basis of the presence of heavy glucose derived ^13^C atoms, such as M+2, M+3 and M+4, M+5 and M+7 (sum of the previous). While glycine and cysteine labeling is expected to be low in the light of their relative abundance in comparison to the light M+0 isotopomers (Suppl. Fig. 4), heavy C atoms might be incorporated from glutamate derived via transamination of labeled ketoglutarate from acetyl-CoA produced via glycolytic consumption of labeled glucose (Suppl. Fig. 6A) (J-L). Isotopomer distribution of glutathione in glutamine labeling experiments. Grouped columns in the left indicate TAp63α-expressing (doxycyline supplemented cells – Dox +), while columns in the right refer to non-induced controls (Dox -). Isotopomers are distributed as follows: M+5+1 (fully labeled glutamate in both carbon and nitrogen atoms), M+5 (glutamate labeled only in carbon atoms). M+2 and M+3 (fast equilibrium with ketoglutarate; M+4 and 4+1 isotopomers were not detected). Kinetics assays have been performed by harvesting cells at 1, 6, 12 and 24h from medium replacement (which in turn was performed at 24h from doxycyline supplementation). From left to right, the graphs represent only heavy isotopomers (J), all isotopomers (K), and only heavy isotopomers in Dox + against Dox – cells at 1h after medium replacement (L). In L, differential results were statistically significant (*p < 0.01* Student's t-test). Kinetics assays have been performed by harvesting cells at 1, 6, 12 and 24h from medium replacement (which in turn was performed at 24h from doxycyline supplementation). * and # indicate statistical significance for inter-group (Dox + vs Dox -) or intra-group variations (time-course analyses). The number of * and # is related to *p-values* < 0.05; 0.01 or 0.001, respectively.

Recently, p73-dependent serine biosynthesis has been coupled to GSH homeostasis in cancer[22,24]. Owing to relative abundances (Suppl. Fig. 4), GSH incorporation of heavy isotopomers from glycine and cysteine was expected to be negligible. On the other hand, isotopomer distribution of GSH in ^13^C-glucose experiments was consistent with the progressive incorporation of heavy labeled glutamate (Fig. 3A, Suppl. Fig. 4). Overall, minor albeit significant heavy isotopomer incorporation in GSH was observed in induced cells during time-course analyses (Fig. 3A – from glucose; D - from glutamine), despite total GSH being comparable to non-induced controls (Fig. 3B,E). On the other hand, glutamate incorporation from labeled glutamine in GSH was higher in induced cells in comparison to controls only before reaching the steady state (<6h from medium replacement - Fig. 3F). TAp63α-dependent glutamate utilization in GSH biosynthesis suggests that glutamate might represent a limiting factor driving TAp63α-mediated responses to oxidative stress, accumulating in the form of GSSG in non-induced cells (Fig. 3C). Together with the up-regulation of the NADPH-generating PPP, these results are suggestive of a likely antioxidant effect mediated by TAp63α.

### TAp63α expression decreases the fueling of glucose and glutamine in Krebs cycle

**Figure 4:**
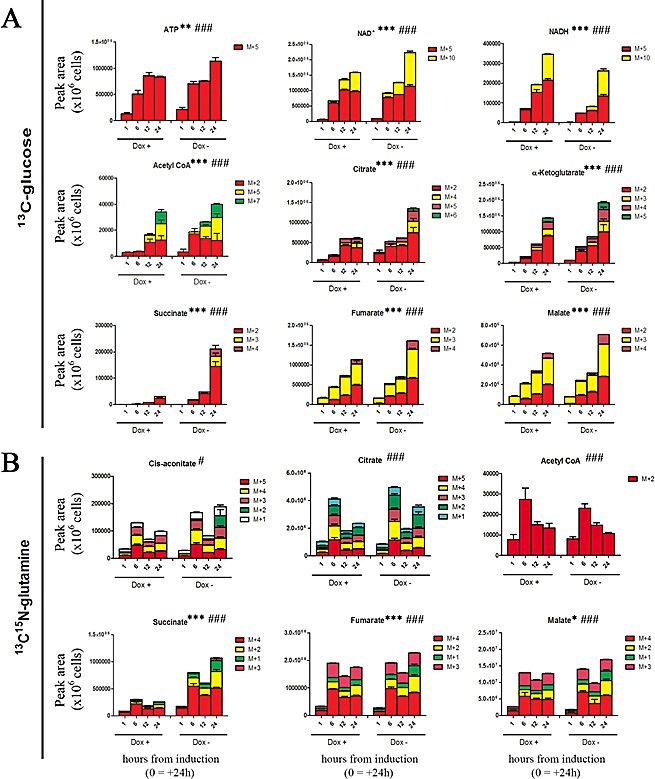
Effect on Krebs cycle (heavy labeled isotopomers only) (A) Distribution of heavy isotopomers of metabolites of the Krebs cycle from ^13^C-glucose labeling experiments, graphed from left to right, from top to bottom in their order of appearance in the oxidative reactions of the TCA cycle pathway. Isotopomers are indicated as M+5 (incorporating five ^13^C atoms) or M+10 (for nucleotides deriving one or two ribose moieties from heavy glucose), M+2, M+3, M+4, M+5, M+6 (progressively heavy labeled Krebs cycle intermediates from glucose-derived acetyl-CoA). (B). Distribution of heavy isotopomers of metabolites of the Krebs cycle from ^13^C^15^N-glutamine labeling experiments. Isotopomers are distributed as follows: M+4 (incorporating four ^13^C atoms, i.e. fully labeled from glutamine-derived ketoglutarate), M+2 and +1 (subsequent oxidative cycles of the TCA supplied by other unlabeled carbon sources than labeled glutamine). M+3 isotopomers of succinate, fumarate and malate would derive from reductive carboxylation of ketoglutarate. Grouped columns in the left indicate TAp63α-expressing (doxycyline supplemented cells – Dox +), while columns in the right refer to non-induced controls (Dox -). Kinetics assays have been performed by harvesting cells at 1, 6, 12 and 24h from medium replacement (which in turn was performed at 24h from doxycyline supplementation). * and # indicate statistical significance for inter-group (Dox + vs Dox -) or intra-group variations (time-course analyses). The number of * and # is related to *p-values* < 0.05; 0.01 or 0.001, respectively.

Defects of Krebs cycle enzymes and a reduced rate of oxidative phosphorylation are tied to cancer, as they can contribute to tumor progression or suppression [35]. In glucose labeling experiments, heavy labeling of Krebs cycle precursors and intermediates (including acetyl-CoA, citrate, α-ketoglutarate, succinate, fumarate and malate) was reduced upon TAp63α overexpression (Fig. 4A). However, the total levels of Krebs cycle intermediates did show statistically significant changes only in early time points (Suppl. Fig. 4A). In late time points, light isotopomers compensated for the lower amounts of heavy labeled counterparts in TAp63α expressing cells (Suppl. Fig. 6A). This was with the exception of succinate. Indeed, succinate level was highly altered in the oxidative arm of the Krebs cycle in response to the induced expression of TAp63α (Fig. 4A). In the light of these results, we next hypothesized that some other carbon sources, such as glutamine, could progressively fuel Krebs cycle upon induction of TAp63α. Indeed, p63 is also known to promote the expression of glutaminase 2 [36], the metabolic enzyme responsible for the conversion of glutamine in glutamate, and also therefore partially responsible of the control of anaplerotic flux. However, in glutamine labeling experiments, no statistically significant alterations were observed in intracellular total and heavy glutamine levels (Suppl. Fig. 7A,B), as well as in glutamine conversion to glutamate (heavy isotopomers - Supp Fig. 7C-D). On the other hand, utilization of labeled-glutamate to produce α-ketoglutarate was more sustained in control cells than in TAp63α expressing cells (Suppl. Fig. 7E,F). Also glutamine-labeling experiments showed a substantial decrease of succinate levels in TAp63 expressing cells (Fig. 4B, Suppl. Fig. 6B), which might indicate that less glutamine is committed to the TCA cycle, consistently with lower labeling of TCA intermediates downstream to alpha-ketoglutarate (Fig. 4B). Alternative explanations imply that decreased levels of succinate might either result from a blockade of the upstream oxoglutarate dehydrogenase or succinyl-CoA ligase, or rather suggest an alternative fate for succinate. Downstream to succinate, reduction in fumarate and malate levels showed minor, albeit statistically significant, deviations from control levels (Fig. 4B). Isotopomer distribution in ^13^C-glucose experiments suggests that oxidative Krebs cycle intermediates down-stream to ketoglutarate might be fluxed from other precursors in addition to glutamate, or rather be produced as a result of reductive carboxylation. Indeed, glutamate-derived ketoglutarate can either enter in to oxidative Krebs cycle or rather serve as a substrate for reductive carboxylation reactions [37], thus enabling the cells to replenish acetyl-CoA reservoirs for anabolic purposes, such as fatty acid biosynthesis (Suppl. Fig. 8). Although TAp63 overexpressing cells showed reduction of glutamine-derived alpha-ketoglutarate, they presented similar levels of reductive carboxylation-derived isotopomers (+5 citrate and aconitate, +3 malate and fumarate, +2 acetyl-CoA). However, it is worth noting that these isotopomers can accumulate without net reductive carboxylation flux, even in hypoxic cells or cells with IDH mutations [38,39]. While total ATP levels did not change between doxycyline-supplemented and untreated cells, induced expression of TAp63α corresponded to a faster accumulation of the reduced form of the coenzyme nicotinammide adenine dinucleotide (NADH) (Suppl. Fig. 4A). NADH is a key marker of energy metabolism, and its accumulation is an indirect indicator of the glycolsys/mitochondrial metabolism [40], as it either accompanies malignant transformation or the up-regulation of PPP and serine biosynthesis at the expenses of lactic fermentation [41].

### TAp63α might influence fatty acid synthesis

Recent literature suggests a role for p63 in the promotion of fatty acid synthesis and depression of beta-oxidation by transcriptionally activating Sirt1, AMPKα2, and LKB1 [26]. By monitoring labeled acetyl-CoA, either derived from glucose or glutamine, we investigated fatty acid biosynthesis rates [37], hereby determined by analyzing the isotopomeric distribution of palmitate. As a result, glutamine-derived acetyl-CoA was more rapidly fluxed toward palmitate anabolism in TAp63α expressing cells in comparison to non-induced controls (Suppl. Fig. 9A,B). However, it is worth noting that labeled palmitate was but a minor percentage of the total palmitate (Supp. Fig 9 C,D), and that fatty acid labeling from glutamine in hypoxia can be explained by spreading of label without net reductive IDH flux [38].

### TAp63-dependent regulation of metabolism represents a prognostic factor in cancer

**Figure 5:**
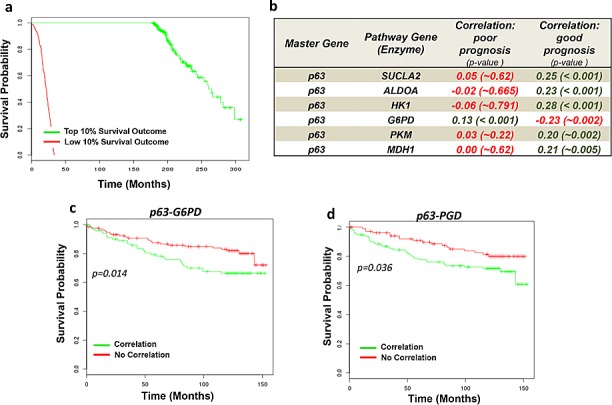
Bioinformatic validation of p63 in cancer metabolism (A) Survival estimation curves of top 10% (good prognosis) and low 10% (bad prognosis) survival outcome from Metabric dataset. Each cohort includes approximately 200 samples. (B) Table showing correlation factors between p63 and metabolic enzymes of interest in the two cohorts “good prognosis” and “bad prognosis”. In dark green positive correlation, in red negative correlation. (C-D) Positive p63/G6PD and p63/PDG correlations (“Correlation” subgroups, in green) represent a negative prognostic factor for survival of breast cancer patients. Breast cancer datasets (GSE3494) was split in two cohorts ‘p63/metabolic enzyme Interaction’ and ‘p63/metabolic enzyme NO Interaction’ on the basis of existence or not of their correlation. Survival estimation was compute to show prediction of survival outcome for the two cohorts.

We next evaluated the clinical relevance in human cancers of TAp63-dependent regulation of cellular metabolism. By using a bioinformatic approach we implemented a series of coexpression analysis with patient survival estimation in human breast cancer datasets. Assessment of the impact on prognosis of correlation between p63 and metabolic enzymes would suggest a clinical relevance of this molecular axis in human cancers. First we used a big dataset from METABRIC (Molecular Taxonomy of Breast Cancer International Consortium). METABRIC dataset is a collection of over 2,000 clinically annotated primary fresh-frozen breast cancer specimens subjected to copy number and gene expression profiling [42]. The survival annotation of the patients was used to select two cohorts of patients. Top 10% patients with the best-observed survival parameters were selected as “good prognosis” subset (~200 samples) while low 10% with the worst survival records were selected as “poor prognosis” subset (~200 samples) (Fig. 5A). Next, we computed Pearson correlation coefficient between TP63 expression profile and profiles of genes from the PPP for each subset of patients (“good” and “poor” prognosis). Our analysis demonstrated that in patients with non-aggressive cancer we observe significant correlation of mRNA expression between p63 and most genes from the pathways while in patients with aggressive cancer this link is absent (Fig. 5B). Interestingly, the only exception was for glucose-6 phosphate dehydrogenase (G6PD), whose correlation with p63 was mainly identified in bad prognosis subgroup (Fig. 5B). G6PD is the rate-liming step of PPP, converting glucose-6-phosphate to 6-phosphate-gluconolactone. The PPP is a major glucose metabolic pathway required for cellular demands of anabolism and antioxidant defense. PPP indeed has emerged among the major metabolic pathways involved in malignancies. Hence we decided to investigate deeper whether the TAp63-dependent regulation of this pathways showed a relevant function for cancer prognosis. We selected an additional human breast cancer dataset and we split the samples in two cohorts. The first cohort included all the samples to maximize positive correlation between p63 and the metabolic enzyme, while in the second cohort all the other samples were included. This clusterization divided the dataset in two biological groups: “Correlation” and “No Correlation”. Then, we computed survival estimation for the two cohorts, thus estimating the prognostic ability of the gene correlation. This algorithm was applied to the clinical datasets to assess p63 correlation with the major enzymes of PPP (Glucose-6-phosphate dehydrogenase, G6PD and 6-phosphogluconate dehydrogenase, PGD). As shown in Figure 5C,D, the analysis revealed that the existence of p63 correlation with G6PD, PGD, predicted negative prognosis in breast cancer. These analyses revealed that cancer cell might benefit from the existence of p63-dependent regulation of this metabolic pathway. Although bioinformatics analyses did not discriminate among p63 isoforms, the importance of PPP and serine biosynthesis for the maintenance of redox balance, was also confirmed by the hereby observed increased GSH level in TAp63 expressing cells, suggested that TAp63 might support cell redox homeostasis by promoting their cellular antioxidant defense.

In summary, the present study indicates that TAp63α induces a wide series of metabolic consequences (Suppl. Fig. 10). Induced expression of TAp63α in SaOs-2 cells boosted cell glycolysis and reduced glucose derivative channeling to the Krebs cycle. By promoting the accumulation of early glycolytic intermediates, TAp63α favored the glycolytic branching towards the PPP, which was uncoupled from nucleotide biosynthesis albeit prevented GSSG accumulation. Results also suggested that TAp63α might either promote a lower consumption or a faster rate of serine biosynthesis, which however did not correspond to increased leves of labeled glycine and cysteine. TAp63α depressed glutamine-derived glutamate utilization to fuel catabolic reactions in the TCA cycle. However, from the present results we could observe only minor Tap63α-dependent increases of glutamine-derived labeling in palmitate, though it is unclear whether this could be tied to increased rates of reductive carboxylation and *de novo* synthesis of fatty acids. These findings highlight the importance of TAp63 for the maintenance of antioxidant defense and contribute to elucidate the role of TAp63 in cellular metabolism.

## METHODS

### Cell culture and reagents

Human osteosarcoma SAOS-2 with doxycycline-inducible expression of HA-TAp63α were cultured in Dulbecco's modified Eagle's medium (Gibco, Invitrogen), supplemented with 10% fetal bovine serum (FBS), 100 μg/ml penicillin and 100 μg/ml streptomycin (all Gibco, Invitrogen) and cultured at 37 C° with 5% CO_2_. To induce TAp63α expression, Saos-2- TAp63α cells were grown in the presence of doxycyclin (2.5 μg/ml) for 12, 24 and 48 hours as previously reported [27].

### Metabolomics experiments

Saos-2 cells (0.5×10^6^) were seeded in triplicate wells of 6-well plates in high glucose DMEM supplemented with supplemented with 10% fetal bovine serum (FBS), 100μg/ml penicillin and100 μg/ml streptomycin (Gibco, Invitrogen) and 2mM L-glutamine. Duplicate plates were seeded for cell counts. After 24 h from doxycyline supplementation to the medium (2.5μg/ml), cells were washed with PBS and received heavy ^13^C-glucose or ^13^C^15^N-glutamine-substituted media (Cambridge Isotopes).

Extraction of metabolites was performed at 1, 6, 12 and 24h upon supplementation of heavy labeled media (0h = 24h from TAp63α induction via doxycycline supplementation). Cells were washed with PBS and metabolites extracted using methanol/acetonitrile/dH2O (5:3:2) (1–2×10^6^ cells per ml). Samples were vortexed for 30minutes at 4°C and thus centrifuged at 16000g for 15min at 4°C. Supernatants were collected for subsequent analyses. MS analyses were performed in polarity switch mode on an Orbitrap Exactive (Thermo Scientific) in line with an Accela autosampler and an Accela 600 pump (Thermo Scientific). Column hardware consisted of a Sequant ZIC-pHILIC column (2.1×150mm, 5μm) coupled to a Sequant ZIC-pHILIC guard column (2.1×20mm, 5 μm) (Merck). Flow rate was 100 ml min^−1^, buffers consisted of acetonitrile (ACN) for A, and 20mM (NH_4_)_2_CO_3_, 0.1% NH_4_OH in ddH_2_O for B.

Gradient ran from 80% to 40% ACN in 20min, followed by a wash at 20% ACN and re-equilibration at 80% ACN. Metabolites were identified and quantified using LCquan software (Thermo Scientific). Metabolites were positively identified on the basis of exact mass within 5 p.p.m., further validated by concordance with standard retention times and plotted as the peak area for each metabolite. Isotopomer distributions were determined on the basis of the expected reactions involving the investigated metabolite and by looking the actual isotopomeric distribution of mass spectra chromatograms. Results were graphed with Graphpad Prism 5.01 (Graphpad Software Inc) as means + SD. Statistical analyses were performed with the same software, as a result of paired t-test or two-way ANOVA among the results obtained from doxycycline supplemented (dox+) and non-supplemented (dox-) cells at the steady state or in a time-dependent fashion (independent variables being doxycycline supplementation and time-course assays).

### Immunoblot analysis, antibodies and cell cycle analysis

Immunoblot analysis was performed using whole cell extracts obtained by lysing cell pellets with Triton Buffer (, supplemented with protease and phosphatase inhibitors (Sigma). Proteins were separated by SDS-PAGE (either 8 or 10%, depending on the MW range of the assayed protein), transferred onto PVDF membranes and blocked with TBS buffered saline and 0,1%Tween-20, containing 5% non-fat dry milk for one hour at room temperature (RT). The incubation with primary antibodies was performed for two hours at RT. Antibodies used included: anti-HA (mouse, 1:1000; Clone 16B12 N. MMS101P - Covent), anti-beta actin (mouse, 1:2000; Clone C4 (Millipore) Lot n. 2065987), and anti-p21 (goat, 1:1000; p21 (c-19) sc-397-G - Santa Cruz). Incubation with the appropriate secondary antibody either included LICOR secondary Ab: anti-mouse IgG (donkey, 1:20000; green (800CW) - LT 926-32212 Lot no. C10707-01 (Odyssey)), anti-mouse IgG (donkey, 1:20000; red (IRDye 680) - LT 926-68022 Lot no. C10921-02 (Odyssey)), anti-rabbit IgG (donkey, 1:20000; green (800CW) - LT 926-32213 Lot no. C11020-03 (Odyssey)). Detection was performed with a LICOR scanning apparatus (Odyssey).

### Cell cycle Analysis

Quantification of sub-G1 population and cell cycle phase was performed by FACS analysis of propidium iodide-staining nuclei, carried out in a FACScan flow cytometer (Becton Dickinson, Heidelberg, Germany) using the CELLQuest software system. For sub-G1 analysis, cells were grown and detached from plates via trypsinization, then fixed and stained with propidium iodide (PI, 50mg/ml) for 30 min. For cell-cycle analysis BrdU (Invitrogen) was added to live cells for 100min and detected as described previously [27]. Flow cytometry was performed on a FACSCalibur cytometer. For PI exclusion, PI was added to cell culture medium (1 mg/ml) for 5min. Non-adherent and adherent cells were collected and analysed by Flow cytometry on a FACSCalibur cytometer.

### Bioinformatics analyses

For correlation analysis between p63 and metabolic enzymes in top and low 10% patients from Metabric dataset, we computed Pearson correlation coefficient between p63 expression profile and profiles of genes from the pathway for each subset of patients (“good” and “poor” prognosis). The p-values were computed using Monte-Carlo simulation procedure. The null hypothesis that was tested for each genes pair assumed correlation of expression between p63 and a gene from the pathways (next referred to as a gene P) in a cohort of selected samples to be equal to the correlation in the whole set of samples. To estimate the p-value, a random cohort of samples has been sampled 1000 times and correlation between p63 and a gene P has been computed. Therefore, we deduced a distribution (of size 1000) of correlation between p63 and a gene P in a randomly selected cohort of ~200 samples. The values in the distribution are compared to the value of correlation in the selected cohort of patients and p-value is computed. For example, for pair p63 and SUCLA2 the estimated p-value is < 0.001 which indicates that no one time in 1000 simulations the correlation between p63 and SUCLA2 in the randomly selected cohort of patients has been higher than 0.25 which is correlation between p63 and SUCLA2 in “good prognosis” cohort of patients.

For the survival estimation analysis in p63/G6PD or p63/PDG positive and negative groups we computed correlation between of p63 and enzymes expression profiles in cohort 1 as well as compute the changes in correlation if one sample from the cohort would be removed. The sample with maximal effect on correlation (maximal increase in positive correlation) is moved from cohort 1 to cohort 2. The same procedure is repeated next until there would be no one sample which could be removed from cohort 1 so to increase positive correlation between p63 and the metabolic enzymes. The separation of patients into “cohort 1” and “cohort 2” along with survival information is next used to find any statistical differences in survival outcome. The R statistical package is used to perform survival analyses and to draw KMplots.

## Supplementary Material Figures


