# Supplementary material for: Metabolic effect of TAp63α: enhanced glycolysis and pentose phosphate pathway, resulting in increased antioxidant defense
Source: Oncotarget. 2014 Jul 31;5(17):7722–33. doi: 10.18632/oncotarget.2300 (PMC4202156; doi:10.18632/oncotarget.2300)
Supplement: Supplementary file 1 [file oncotarget-05-7722-s001.pdf]

Metabolic effect of TAp63α: enhanced glycolysis and pentose phosphate pathway, resulting in increased antioxidant defense

Supplementary Material

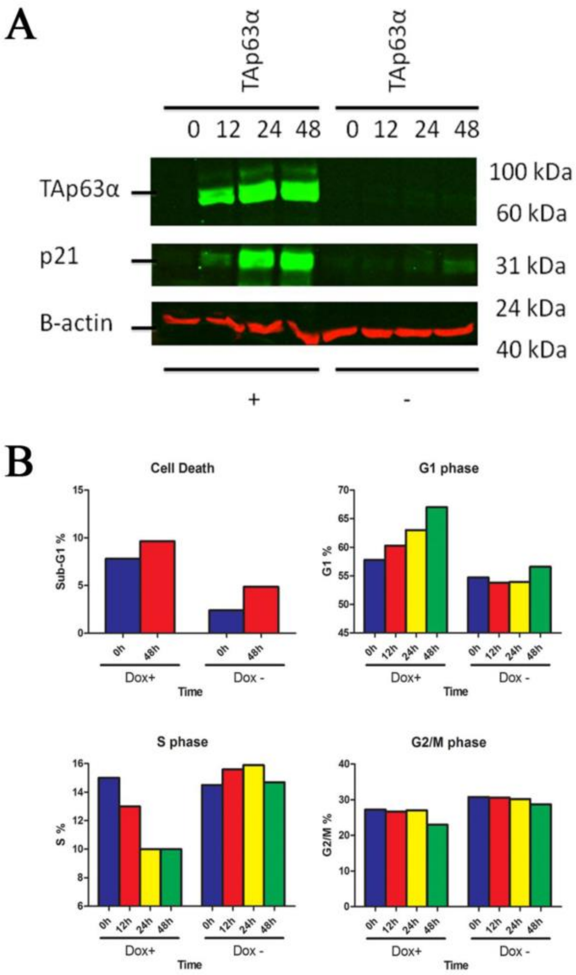

Supplementary Figure 1: Induced expression of TAp63α and cell cycle arrest at G1 phase In **A,** Western blot (LICOR) analysis of doxycyline supplemented (2.5 µg/ml) (+) Tet-On Saos-2 cells against untreated controls (-) to assess the time dependent expression (at 0, 12, 24 and 48h upon doxycyclin addition to the medium) of TAp63α (HA tag) and its target p21. Beta-actin (red) was used as an internal control. In **B**, cell cycle arrest in G1 phase and decrease in S phase were observed progressively upon Tap63α induction (Dox +) as gleaned through FACScan analysis.

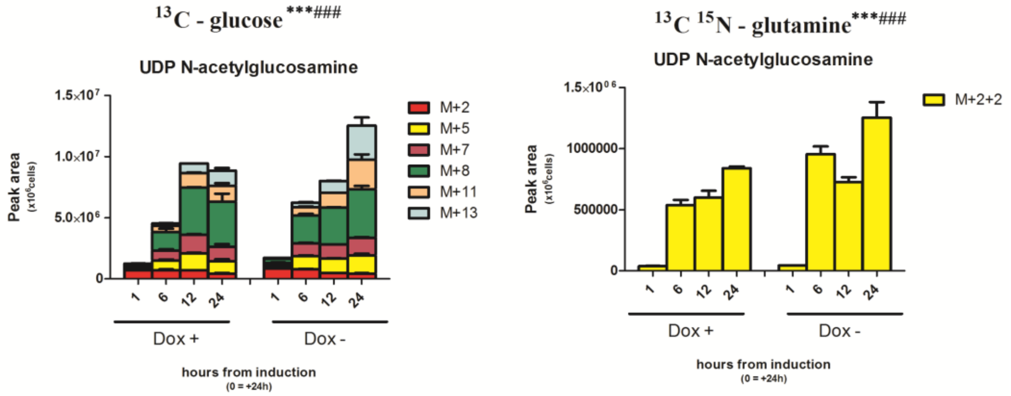

Supplementary Figure 2: Effect on the hexosamine pathway Isotopomer distribution of a representative late metabolite of the Hexosamine Pathway, UDP-N-acetyl glucosamine, both in ^{13}C-glucose (left) and ^{13}C^{15}N-glutamine (right) labeling experiments. Grouped columns in the left indicate TAp63α-expressing (doxycyline supplemented cells – Dox +), while columns in the right refer to non-induced controls (Dox -). Only heavy isotopomers are indicated, as to highlight the variations in the metabolic fluxes only upon medium replacement. For glucose experiments, the M+2 isotopomers incorporate heavy-labeled acetyl groups; M5 incorporate 5 heavy ^{13}C atoms in the ribose moiety; M+7, +8, 11 or 13 correspond to different combinations of the other isotopomers (either including or not a heavy labeled 13C glucosamine moiety). For glutamine experiments, the M+2+2 isotopomer refers to the incorporation of 2 ^{15}N atoms (one in glucosamine and one in uridine) and 2 carbon atoms (acetyl moiety) from heavy glutamine. Kinetics assays have been performed by harvesting cells at 1, 6, 12 and 24h from medium replacement (which in turn was performed at 24h from doxycyline supplementation). * and # indicate statistical significance for inter-group (Dox + vs Dox -) or intra-group variations (time-course analyses). The number of * and # is related to *p-values* < 0.05; 0.01 or 0.001, respectively.

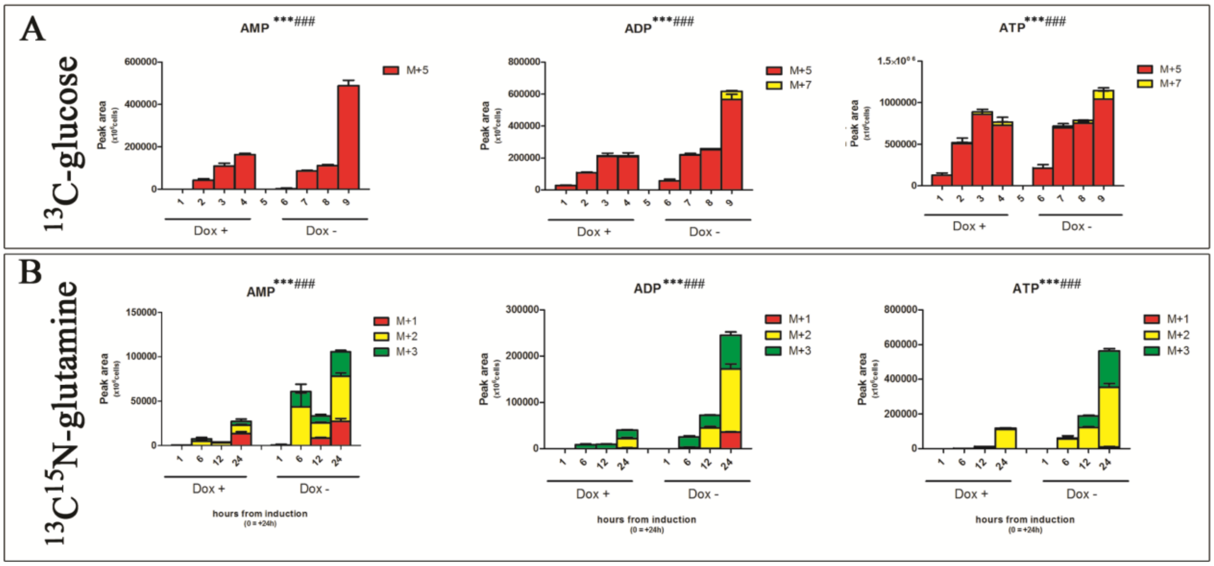

Supplementary Figure 3: Isotopomer distribution of adenosine nucleotides Grouped columns in the left indicate TAp63α-expressing (doxycyline supplemented cells – Dox +), while columns in the right refer to non-induced controls (Dox -). (**A)** Heavy isotopomers from 13C-glucose experiments are reported on the basis of the presence of heavy glucose derived carbon atoms, such as M+5 (ribose moiety, most abundant), M+7 (ribose moiety + 2 glucose-derived carbon atoms from glycine). (**B)** Heavy isotopomers of adenosine-derived nucleosides as assayed during 13C15N -glutamine experiments. Isotopomers are reported on the basis of the presence of heavy glutamine derived nitrogen atoms, such as M+1 and +2 (glutamine-derived nitrogens) or M+3 (M+2, plus a third nitrogen atom from labeled aspartate). Kinetic assays have been performed by harvesting cells at 1, 6, 12 and 24h from medium replacement (which in turn was performed at 24h from doxycyline supplementation). * and # indicate statistical significance for inter-group (Dox + vs Dox -) or intra-group variations (time-course analyses). The number of * and # is related to *p-values* < 0.05; 0.01 or 0.001, respectively.

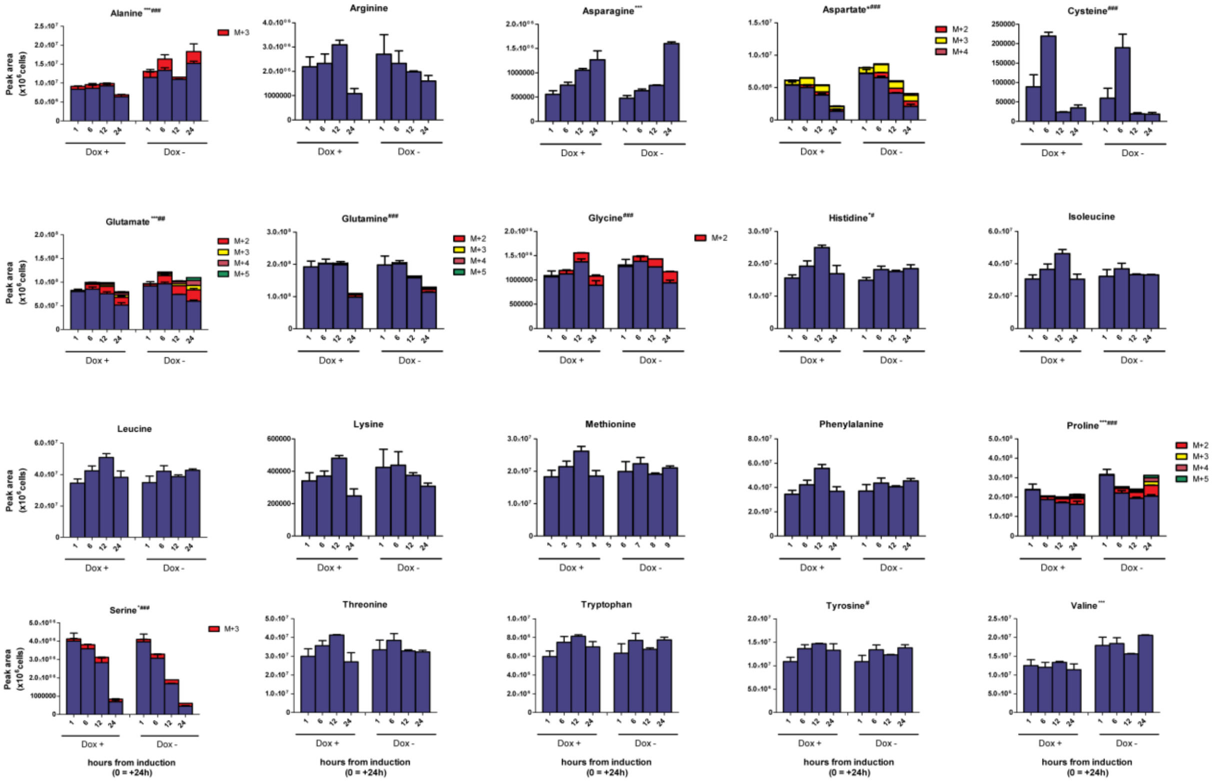

Supplementary Figure 4: Aminoacids Quantitative analyses of aminoacids in doxycyline-supplemented (dox +) or non-supplemented (dox-) cells, at 1, 6, 12 and 24h from medium replacement with heavy labeled glucose (24h after doxycyline addition to the medium). Light isotopomers are graphed (which derive from the uptake of unlabeled aminoacids from the medium or proteolysis) together with heavy isotopomers from ^{13}C-glucose labeling experiments. * and # indicate statistical significance for inter-group (Dox + vs Dox -) or intra-group variations (time-course analyses). The number of * and # is related to *p-values* < 0.05; 0.01 or 0.001, respectively.

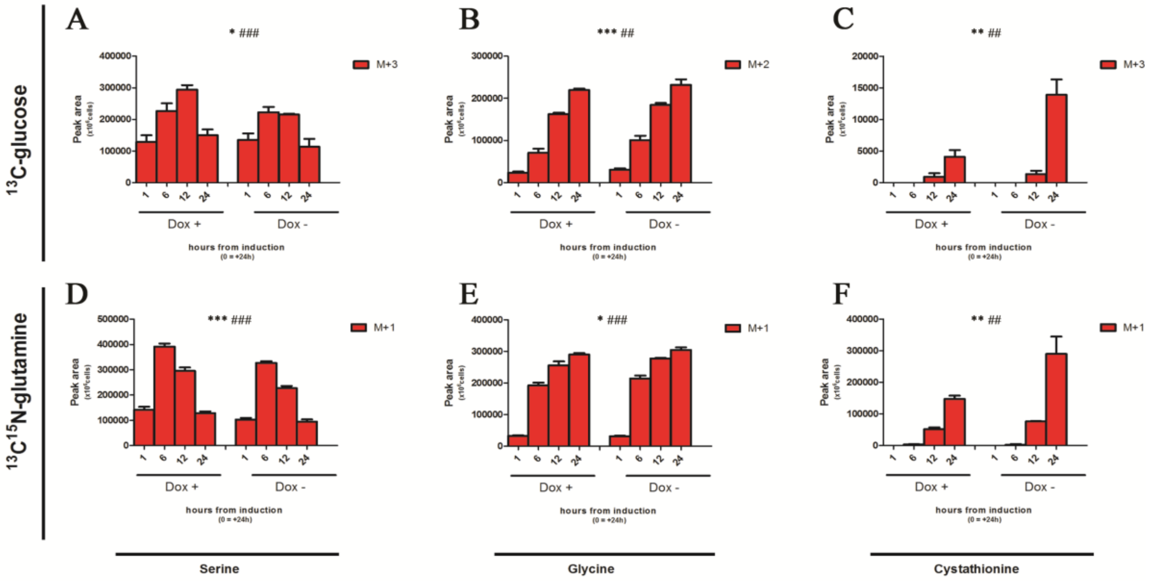

Supplementary Figure 5: Effect on serine biosynthesis and GSH homeostasis (A-F) Heavy isotopomers of serine (**A** and **D**), glycine (**B** and **E**) and cystathionine (**C** and **F**), as derived from *de novo* biosynthesis. The top row indicates the results from ^{13}C-glucose labeling experiments, whereby serine (M+3 - **A**), glycine (M+2 - **B**) and cystathionine (M+3 - **C**) derived heavy-labeled ^{13}C atoms from 3-phosphoglycerate. The bottom row refers to serine, glycine and cystathionine (M+1 – **D-F**) deriving their ^{15}N atom from labeled heavy glutamine. Grouped columns in the left indicate TAp63α-expressing (doxycyline supplemented cells – Dox +), while columns in the right refer to non-induced controls (Dox -).

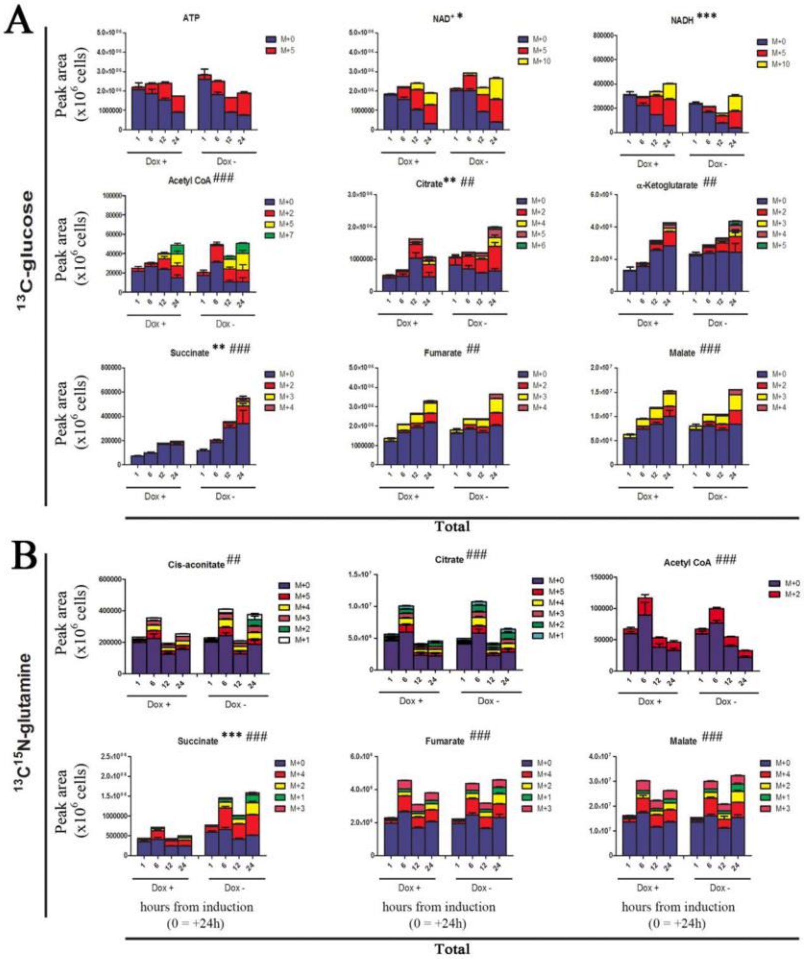

Supplementary Figure 6: Effect on Krebs cycle (A) Isotopomer distribution of metabolites of the Krebs cycle from ^{13}C-glucose labeling experiments, graphed from left to right, from top to bottom in their order of appearance in the oxidative reactions of the TCA cycle pathway. Isotopomers are indicated as M+0 (light, no ^{13}C atom incorporated), M+5 or M+10 (for nucleotides deriving one or two ribose moieties from heavy glucose), M+2, M+3, M+4, M+5, M+6 (progressively heavy labeled Krebs cycle intermediates). **(B)** Isotopomer distribution of Krebs cycle intermediates in glutamine labeling experiments. Both heavy and light isotopomers are graphed. Isotopomers are distributed as follows: M+0 (light, no ^{13}C atom incorporated), M+4 (fully labeled from glutamine-derived ketoglutarate), M+2 and +1 (subsequent oxidative cycles of the TCA supplied by other unlabeled carbon sources than labeled glutamine). M+3 isotopomers of succinate, fumarate and malate would derive from reductive carboxylation of ketoglutarate. Grouped columns in the left indicate TAp63α-expressing (doxycyline supplemented cells – Dox +), while columns in the right refer to non-induced controls (Dox -). Kinetics assays have been performed by harvesting cells at 1, 6, 12 and 24h from medium replacement (which in turn was performed at 24h from doxycyline supplementation). * and # indicate statistical significance for inter-group (Dox + vs Dox -) or intra-group variations (time-course analyses). The number of * and # is related to *p-values* $<$ 0.05; 0.01 or 0.001, respectively.

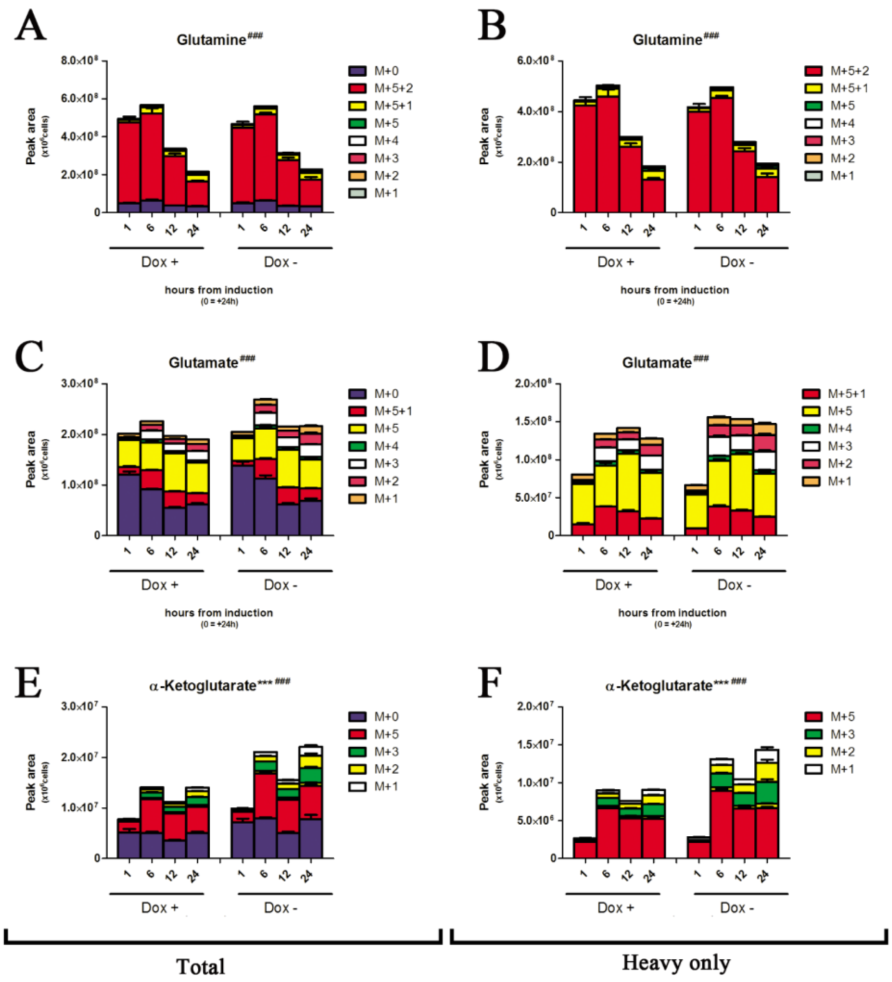

Supplementary Figure 7: Effect on glutamine conversion to glutamate and metabolism through the Krebs cycle Isotopomer distribution of glutamine (**A, B**) and glutamine-derived glutamate (**C**, **D**) and alpha-ketoglutarate (**E**, **F**) in $^{13}C^{15}N$-glutamine labeling experiments. Grouped columns in the left indicate TAp63α-expressing (doxycyline supplemented cells – Dox +), while columns in the right refer to non-induced controls (Dox -). Isotopomers are distributed as follows: M+5+2 (labeled glutamine in both carbon and nitrogen atoms); M+5+1 (fully labeled glutamate), or M+5 (produced by transamination of fully labeled ketoglutarate via the introduction of one unlabeled nitrogen atom from aspartate); and M+4, +3, +2, +1 (fully labeled ketoglutarate) and relative isotopomers deriving from the incorporation of unlabeled carbon atoms at each next cycle of the Krebs cycle (if fueled by other carbon sources, such as glucose). Kinetics assays have been performed by harvesting cells at 1, 6, 12 and 24h from medium replacement (which in turn was performed at 24h from doxycyline supplementation).). * and # indicate statistical significance for inter-group (Dox + vs Dox -) or intra-group variations (time-course analyses). The number of * and # is related to *p-values* < 0.05; 0.01 or 0.001, respectively.

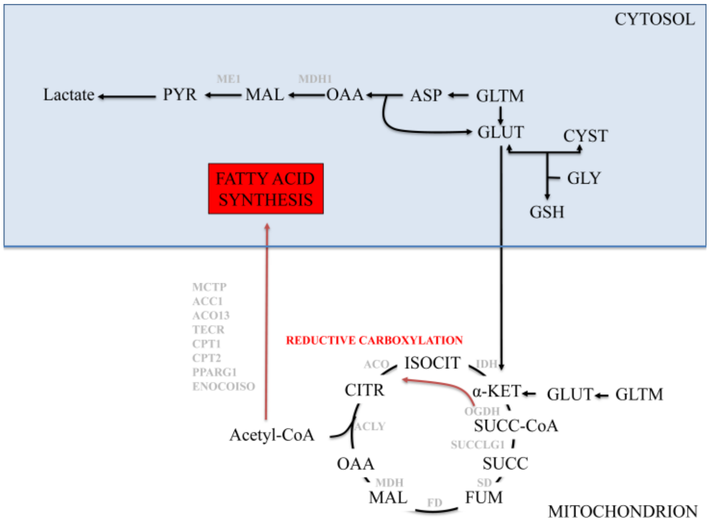

Supplementary Figure 8: A simplified overview of metabolites and enzymes involved in Krebs cycle, reductive carboxylation and fatty acid biosynthesis

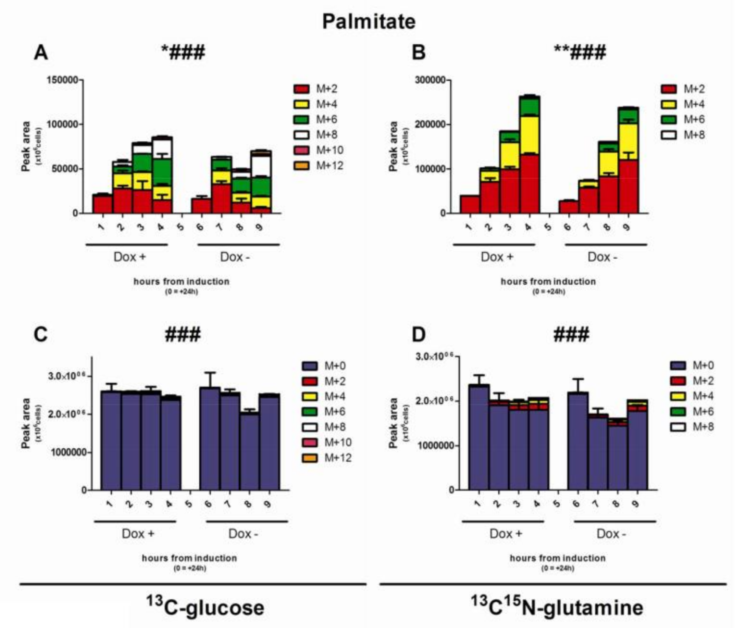

Supplementary Figure 9: Effect on fatty acid synthesis Isotopomer distribution of a representative late metabolite of fatty acid synthesis, the C16:0 fatty acid palmitate, both in ^{13}C-glucose (left) and ^{13}C^{15}N-glutamine (right) labeling experiments. Grouped columns in the left indicate TAp63α-expressing (doxycyline supplemented cells – Dox +), while columns in the right refer to non-induced controls (Dox -). In **A** and **B,** only heavy isotopomers are indicated, as to highlight the variations in the metabolic fluxes only upon medium replacement. In **C** and **D** all isotopomers (light and heavy) are shown. In both the experiments, two ^{13}C atoms (M+2 and relative multiples) are added from labeled acetyl-CoA at incremental steps. Kinetics assays have been performed by harvesting cells at 1, 6, 12 and 24h from medium replacement (which in turn was performed at 24h from doxycyline supplementation). * and # indicate statistical significance for inter-group (Dox + vs Dox -) or intra-group variations (time-course analyses). The number of * and # is related to *p-values* < 0.05; 0.01 or 0.001, respectively.

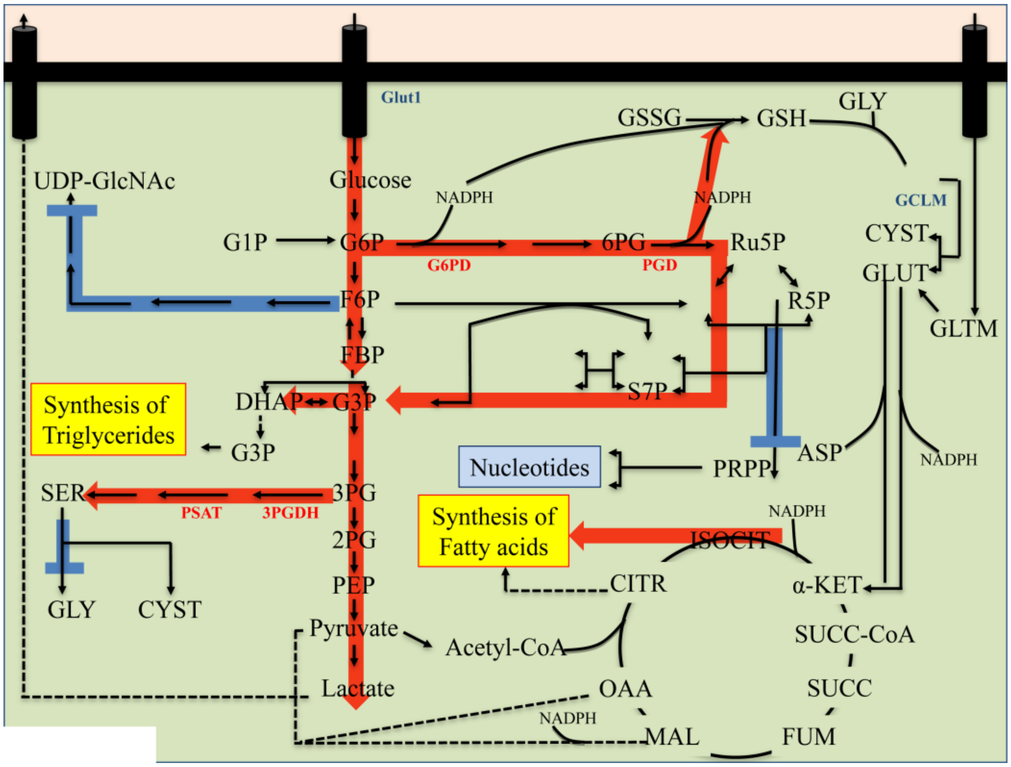

Supplementary Figure 10: Diagram of the metabolic effects of TAp63α An overview of metabolic cycles affected by Tap63α expression, as gleaned from labeling metabolomics experiments. Pentose Phosphate Pathway enzymes glucose 6-phosphate dehydrogenase (G6PD) and phosphogluconate dehydrogenase (PGD) are highlighted in red, together with the serine synthesis pathway enzymes 3-phosphoglycerate dehydrogenase (3PGDH) and phosphoserine aminotransferase (PSAT).
